# Socioeconomic differences in children’s television viewing trajectory: A population-based prospective cohort study

**DOI:** 10.1371/journal.pone.0188363

**Published:** 2017-12-06

**Authors:** Junwen Yang-Huang, Amy van Grieken, Henriëtte A. Moll, Vincent W. V. Jaddoe, Anne I. Wijtzes, Hein Raat

**Affiliations:** 1 The Generation R Study Group, Erasmus Medical Center, Rotterdam, The Netherlands; 2 Department of Public Health, Erasmus Medical Center, Rotterdam, The Netherlands; 3 Department of Pediatrics, Erasmus Medical Center, Rotterdam, The Netherlands; 4 Department of Epidemiology, Erasmus Medical Center, Rotterdam, The Netherlands; 5 Department of Kinesiology, KU Leuven, Leuven, Belgium; McMaster University, CANADA

## Abstract

We aimed to evaluate the association between family socioeconomic status and repeatedly measured child television viewing time from early childhood to the school period. We analyzed data on 3,561 Dutch children from the Generation R Study, a population-based study in the Netherlands. Parent-reported television viewing time for children aged 2, 3, 4, 6 and 9 years were collected by questionnaires sent from April 2004 until January 2015. Odds ratios of watching television ≥1 hour/day at each age were calculated for children of mothers with low, mid-low, mid-high and high (reference group) education and children from low, middle and high (reference group) income households. A generalized logistic mixed model was used to assess the association between family socioeconomic status and child television viewing time trajectory. The percentage of children watching television ≥1 hour/day increased from age 2 to 9 years for all children (24.2%-85.0% for children of low-educated mothers; 4.7%-61.4% for children of high-educated mothers; 17.2%-74.9% for children from low income households; 6.2%-65.1% for children from high income households). Independent socioeconomic effect in child television viewing time was found for maternal educational level. The interaction between net household income and child age in longitudinal analyses was significant (p = 0.01), indicating that the television viewing time trajectories were different in household income subgroups. However the interaction between maternal educational level and child age was not significant (p = 0.19). Inverse socioeconomic gradients in child television viewing time were found from the preschool period to the late school period. The educational differences between the various educational subgroups remained stable with increasing age, but the differences between household income groups changed over time. Intervention developers and healthcare practitioners need to raise awareness among non-highly educated parents that the socioeconomic gradient in television viewing time has a tracking effect starting from preschool age.

## Introduction

Sedentary behaviors, including screen-related behaviors (i.e. watching television [TV] and playing computer / electronic games) and non-screen related behaviors (i.e. reading), are highly prevalent during childhood [1, 2]. As a key children’s sedentary leisure time pursuit, parent-reported screen time behavior has been linked with adverse health outcomes in childhood including obesity [3], cardiovascular diseases and increased metabolic risk [4, 5]. Given the adverse health outcomes, there are recommendations to limit screen time in childhood. The recommendation from the American Academy of Pediatrics limits screen use to 1 hour per day in children 2 to 5 years of age [6]. Australia and Canada government health authority guidelines recommend that children aged 5–12 years should spend no more than 2 hours a day on electronic media for entertainment [7, 8]. Nonetheless, the majority of young children exceed the recommended levels [9, 10]. Although media use has changed over the past decade aided by the increase in video games and mobile phone use, evidence suggests that the most common screen time behavior continues to be TV viewing[2]. Children who watch more TV at young childhood tend to stay high level of TV viewing time in adolescence [11, 12]. Furthermore, a 32-year follow-up study reported that childhood TV viewing time tracked into adulthood [13]. Little is known about the TV viewing time trajectory from early childhood (i.e. preschool age) to late childhood (i.e. school age, early adolescence) [14]. Longitudinal studies evaluating child TV viewing time trajectory may provide important information to policy makers and researchers regarding the optimal timing of preventive interventions aimed to reduce screen time in childhood.

In addition to identifying important periods in the development of TV viewing behavior, it is important to identify those children who are at increased risk of high levels of TV viewing, and that would benefit from interventions the most. The socioeconomic inequalities in TV viewing time have been well documented, but results have been inconsistent [15]. e.g. According to one study, children (aged 6 to 11) of non-highly-educated mothers were more likely to watch more TV than children of highly-educated mothers [16]. Similarly, another study reported that among children aged 8-to-11-years, those from higher socioeconomic status (SES) groups spent less time watching TV than children from low SES groups [17]. On the other hand, a Greek study reported an inverse association between maternal educational level and TV viewing time among children aged 1–2 years but not among children aged 3–5 years [18]. Furthermore, most of the performed studies are cross-sectional in design [15], and little is known about how socioeconomic inequalities in TV viewing time evolve longitudinally [19].

The aims of the present study were threefold. First, we aimed to assess TV viewing time from early childhood (age 2 years) to the school period (age 9 years). Second, we aimed to assess the cross-sectional association between family SES and TV viewing time with data available at 5 points in time (ages 2, 3, 4, 6 and 9 years). Third, we aimed to evaluate the longitudinal association between family SES and child TV viewing time trajectory from child age 2 to 9 years. We hypothesized that child TV viewing time would increase over time, across all socioeconomic subgroups. We hypothesized that the TV viewing time trajectories would be different for socioeconomic subgroups.

## Methods

### Study design

This study was embedded in The Generation R Study, a population-based prospective cohort study from fetal life onwards [20]. Midwifes and obstetricians invited all pregnant women under their care with an expected delivery date between April 2002 and January 2006, living in Rotterdam, the Netherlands, at time of delivery to participate in the Generation R Study. More details on the study design and participant inclusion procedure can be found in the design paper by Jaddoe et al [21]. The study was conducted in accordance with the guidelines proposed in the World Medical Association Declaration of Helsinki and has been approved by the Medical Ethical Committee at Erasmus MC, University Medical Center Rotterdam. Written informed consent was obtained from all participants.

### Study population

Consent for postnatal follow-up during the preschool period (0–4 years) and/or the school period (6 and 9 years) onwards was available for 4432 children of Dutch mothers. Mothers were considered to be Dutch when both of her parents were born in the Netherlands [22]. We excluded children with missing information on television viewing at all measuring time points (n = 467). To avoid clustering of data, we further excluded second (n = 365) and third children (n = 9) of the same mother, leaving a study population of 3561 participants.

### Family socioeconomic status

Our indicators of family SES were maternal educational level and net household income. The highest educational level attained by the mother was collected using questionnaires at enrollment. The Dutch Standard Classification of Education was used to categorize 4 levels of education: high (university or PhD degree) (n = 1164), mid-high (higher vocational training) (n = 929), mid-low (>3 years general secondary school, intermediate vocational training) (n = 911) and low (no education, primary school, lower vocational training, intermediate general school, or 3 years or less general secondary school) (n = 441) [23]. Net household income was assessed using questionnaires at child age 2 years and classified into 3 categories: high (>€3300 per month) (n = 1378), middle (€2000–3300 per month) (n = 1026) and low (<€2000 per month) (n = 352) [24].

### Television viewing time

Parent-reported TV viewing time was assessed at 5 measuring time points (child age 2, 3, 4, 6 and 9 years). The questionnaires were intended for the mother. If the mother was not able to complete the questionnaire, it could be completed by the other parent/caregiver. Parents were asked to indicate the mean duration per day their child spent on TV viewing in a multiple-choice question (i.e. 1–2 hours). Subsequently, they were asked about the average number of days per week or/and weekends their child spent time on TV viewing (i.e. 2 days per week). We assigned the middle number of hours (e.g. 1.5 hours for “1–2 hours”) to each category, as the duration of TV viewing per session. The average TV time per day was derived by multiplying the duration per day by the number of days of TV viewing, which was then divided by seven. Week- and weekend days were combined. However, the number of days of TV viewing was not available at child age 2 and 3 years. Therefore, at age 2 and 3 years we used the number of days of TV viewing at age 4 years when calculating the average TV time per day. At age 4, 51% of parents indicated that their children watched TV seven days per week. Other details on the TV viewing time measures are available in the supplemental material S1 Table. TV viewing time was dichotomized at more than or equal to 1 hour per day according to the latest recommendation from the American Academy of Pediatrics [6]. Sensitivity analyses using a secondary outcome variable dichotomized at 2 hours/day was also performed [7, 8]. Results are available in the supplemental material S2 Table, S1 and S2 Figs.

### Potential confounders

Child’s gender, age, single parenthood (single parent, two parents [not necessarily biological parents]), presence of siblings and maternal age were considered potential confounders in the associations of family SES with child’s TV viewing time. Child’s age was obtained by questionnaires at each measuring time point. Single parenthood and maternal age were obtained by a questionnaire at enrollment. Presence of siblings was assessed by a questionnaire at child age 6 years.

### Statistical analyses

Descriptive statistics were used to describe the study population. The cross-sectional associations between family SES and child dichotomized TV viewing time at each measuring time point were assessed using logistic regression models. Child’s gender and age were included as confounders in the models based on previous literature [15]. Maternal age led to a substantial change in effect estimates (i.e. ≥10% change) and was included in the models as confounder as well [25]. The first model included the indicator of family SES and confounders (i.e. two basic models, one for each indicator). The second model assessed the independent effect of the family SES indicator, adjusting for the other SES indicator and confounders (i.e. one full model). Collinearity between maternal educational level and net household income was evaluated by Spearman’s rho correlation coefficients (r = 0.47). The correlation coefficient did not indicate collinearity (r>0.6) and therefore both variables were included in the full models simultaneously. A multiple imputation procedure was used to impute missing values in the covariates (ranging from 0% to 28.2%, see Table 1) [26]. Five imputed datasets were generated using a fully conditional specified model, based on the relationships between all the variables included in this study. Cross-sectional analyses of the association between indicators of family SES and child dichotomized TV viewing time at each measuring time point were performed on both the non-imputed and imputed datasets and the results were comparable between two datasets. Pooled estimates from these five imputed datasets were used to report odds ratios (ORs) and their 95% confidence intervals (CI). A significance level of p<0.05 was taken to indicate a significant association.

**Table 1:** General characteristics of the study population (n = 3561).

		Total	Missing
		N (%)	N (%)
**Family characteristics**			
Maternal educational level	High	1164 (33.8)	116 (3.3)
	Mid-high	929 (27.0)	
	Mid-low	911 (26.4)	
	Low	441 (12.8)	
Net household income	More than €3300/month	1378 (50.0)	805 (22.6)
	€2000-3300/month	1026 (37.2)	
	Less than €2000/month	352 (12.8)	
Single parenthood	Yes	199 (5.8)	159 (4.5)
	No	3203 (94.2)	
Maternal age	Years (mean, SD)	31.9 (4.4)	0
Siblings	Yes	2670 (82.6)	327 (9.2)
	No	564 (17.4)	
**Child characteristics**			
Child’s exact age	Age 2 years	24.4 (1.1)	658 (18.5)
Months (mean, SD)	Age 3 years	36.5 (1.1)	806 (22.6)
	Age 4 years	48.5 (1.0)	728 (20.4)
	Age 6 years	71.8 (4.8)	262 (7.4)
	Age 9 years	116.2 (3.4)	613 (17.2)
Gender	Girl	1766 (49.6)	0
	Boy	1795 (50.4)	
TV viewing time ≥1 hour/day	Age 2 years	266 (10.0)	913 (25.6)
	Age 3 years	704 (27.5)	1004 (28.2)
	Age 4 years	906 (32.4)	764 (21.5)
	Age 6 years	1652 (52.9)	436 (12.2)
	Age 9 years	1858 (69.8)	900 (25.3)
TV viewing time ≥2 hour/day	Age 2 years	0 (0)	913 (25.6)
	Age 3 years	100 (3.9)	1004 (28.2)
	Age 4 years	154 (5.5)	764 (21.5)
	Age 6 years	361 (11.6)	436 (12.2)
	Age 9 years	643 (24.2)	900 (25.3)

Table is based on non-imputed dataset.

Values are means (SD) for normally distributed continuous variables and frequencies (percentage) for categorical variables.

Generalized logistic mixed models (GLMM) were used to assess the association between family SES and child TV viewing time measured repeatedly at age 2, 3, 4, 6 and 9 years. Family SES indicators were added into the GLMM models separately. The best fitting model structure was: $log\frac{\pi_{ij}}{1-\pi_{ij}}=\beta_{0}+\beta_{1}*family\;SES+\beta_{2}*child\;age+\beta_{3}*child\;age*family\;SES+b_{i}$. In this model, *π*_*ij*_ = probability of watching TV more than or equal to 1 hour/day. The p-value of the interaction between family SES and child age indicated whether socioeconomic differences changed with the age of the child.

Descriptive analyses and cross-sectional analyses were performed using Statistical Package of Social Science (SPSS) version 21.0 for Windows (SPSS Inc, Chicago, IL, USA) and longitudinal models were fitted using package lme4 in R version 3.2.5 for Windows (R Foundation for Statistical Computing).

## Results

### Study population characteristics

Table 1 shows characteristics of the study population. At enrollment, 33.8% of the mothers had a high educational level and 12.8% had a low educational level. Of all children, 12.8% of the children belonged to a family with a low household income and 50.0% of the children belonged to a family with a high household income. The percentage of children watching TV ≥1 hour/day increased from age 2 to 9 years. 10.0% of children watched TV more than or equal to 1 hour/day at age 2 years, while at age 9 years, 69.8% of children watched TV more than or equal to 1 hour/day.

### TV viewing time from early childhood to the school period

Table 2 shows the percentages of children watching ≥1 hour TV/day according to family SES at each age. The percentage of children watching TV ≥1 hour/day increased from age 2 to 9 years for all SES subgroups. The percentage increased from 24.2% to 85.0% for children of low-educated mothers and from 4.7% to 61.4% for children of high-educated mothers. The percentage increased from 17.2% to 74.9% for children from low income households and from 6.2% to 65.1% for children from high income households.

**Table 2:** TV viewing time ≥1 hour/day according to family socioeconomic status (n = 3561).

		TV viewing time ≥1 hour/day N (%)				
		Age 2 years	Age 3 years	Age 4 years	Age 6 years	Age 9 years
Maternal educational level	High	46 (4.7)	165 (17.2)	221 (21.6)	451 (43.8)	553 (61.4)
	Mid-high	65 (8.6)	198 (27.0)	242 (30.7)	405 (48.6)	476 (66.0)
	Mid-low	93 (14.5)	214 (35.0)	281 (40.9)	466 (59.6)	529 (77.0)
	Low	59 (24.2)	115 (51.6)	144 (55.4)	263 (71.1)	238 (85.0)
p-value*		**<0.001**	**<0.001**	**<0.001**	**<0.001**	**<0.001**
Net household income	>€3300/month	79 (6.2)	257 (21.2)	313 (24.4)	568 (46.5)	698 (65.1)
	€2000-3300/month	120 (12.8)	282 (32.8)	344 (36.6)	496 (54.4)	563 (69.9)
	<€2000/month	52 (17.2)	100 (35.7)	142 (46.4)	172 (58.3)	188 (74.9)
p-value*		**<0.001**	**<0.001**	**<0.001**	**<0.001**	**0.004**

Table is based on non-imputed dataset.

*p-value assessed by Chi-square tests.

### Cross-sectional association between family socioeconomic status and child TV viewing time

Children of low-, mid-low-, and mid-high-educated mothers were more likely to watch TV ≥1 hour/day compared to children of high-educated mothers at all ages (all p<0.05) (basic model, Table 3). The OR for TV viewing time ≥1 hour/day for children of low-educated mothers was 6.32 (95% CI: 4.12, 9.67) at age 2 years, 5.20 (95% CI: 3.79, 7.14) at age 3 years, 4.41 (95% CI: 3.29, 5.91) at age 4 years, 3.15 (95% CI: 2.42, 4.11) at age 6 years and 3.90 (95%CI: 2.69, 5.63) at age 9 years. Children of low-, and middle-household income families were more likely to watch TV ≥1 hour/day compared to children of high household income families (all p<0.05). The OR for TV viewing time ≥1 hour/day for children from low income households was 2.88 (95% CI: 1.98, 4.21) at age 2 years, 1.93 (95% CI: 1.46, 2.57) at age 3 years, 2.46 (95% CI: 1.90, 3.17) at age 4 years, 1.60 (95% CI: 1.24, 2.06) at age 6 years and 1.87 (95%CI: 1.37, 2.55) at age 9 years. With both SES indicators in the model (full model, Table 3), independent associations with child TV viewing time were found for maternal educational level at all ages and for net household income only at child age 2 and 4 years.

**Table 3:** Associations of family socioeconomic status with TV viewing time (≥1 hour/day) at each age (n = 3561).

		TV viewing time ≥1 hour/day				
		Age 2 years	Age 3 years	Age 4 years	Age 6 years	Age 9 years
Basic model^*^						
Maternal educational level	High	1	1	1	1	1
	Mid-high	**1.90**	**1.80**	**1.61**	**1.25**	**1.25**
		**(1.28, 2.81)**	**(1.42, 2.28)**	**(1.30, 2.00)**	**(1.04, 1.50)**	**(1.02, 1.54)**
	Mid-low	**3.37**	**2.61**	**2.48**	**1.95**	**2.24**
		**(2.32, 4.91)**	**(2.05, 3.31)**	**(2.00, 3.08)**	**(1.60, 2.37)**	**(1.78, 2.82)**
	Low	**6.32**	**5.20**	**4.41**	**3.15**	**3.90**
		**(4.12, 9.67)**	**(3.79, 7.14)**	**(3.29, 5.91)**	**(2.42, 4.11)**	**(2.69, 5.63)**
Net household income	>€3300/month	1	1	1	1	1
	€2000-3300/month	**2.12**	**1.79**	**1.70**	**1.40**	**1.30**
		**(1.57, 2.87)**	**(1.46, 2.19)**	**(1.39, 2.08)**	**(1.19, 1.65)**	**(1.08, 1.56)**
	<€2000/month	**2.88**	**1.93**	**2.46**	**1.60**	**1.87**
		**(1.98, 4.21)**	**(1.46, 2.57)**	**(1.90, 3.17)**	**(1.24, 2.06)**	**(1.37, 2.55)**
Full model^**^						
Maternal educational level	High	1	1	1	1	1
	Mid-high	**1.68**	**1.68**	**1.47**	1.21	1.23
		**(1.12, 2.51)**	**(1.32, 2.14)**	**(1.18, 1.84)**	(1.00, 1.47)	(0.99, 1.53)
	Mid-low	**2.82**	**2.38**	**2.16**	**1.86**	**2.18**
		**(1.89, 4.22)**	**(1.84, 3.08)**	**(1.71, 2.73)**	**(1.50, 2.32)**	**(1.70, 2.80)**
	Low	**4.94**	**4.69**	**3.58**	**3.01**	**3.69**
		**(3.09, 7.88)**	**(3.32, 6.62)**	**(2.61, 4.91)**	**(2.24, 4.04)**	**(2.49, 4.02)**
Net household income	>€3300/month	1	1	1	1	1
	€2000-3300/month	**1.47**	**1.30**	**1.29**	1.12	1.01
		**(1.06, 2.03)**	**(1.04, 1.62)**	**(1.04, 1.61)**	(0.94, 1.35)	(0.82, 1.24)
	<€2000/month	**1.58**	1.12	**1.56**	1.06	1.16
		**(1.04, 2.39)**	(0.82, 1.52)	**(1.18, 2.07)**	(0.80, 1.41)	(0.83, 1.63)

Table is based on imputed dataset. Bold print indicates statistical significance. Values represent odds ratios and 95% confidence intervals derived from multiple logistic regression analyses.

* Adjusted for confounders (i.e. child's gender and exact age at measurement and maternal age at enrollment).

** Additional adjusted for the other family socioeconomic status indicators.

### Longitudinal association between family socioeconomic status and the child TV viewing time trajectory

**Fig 1:**
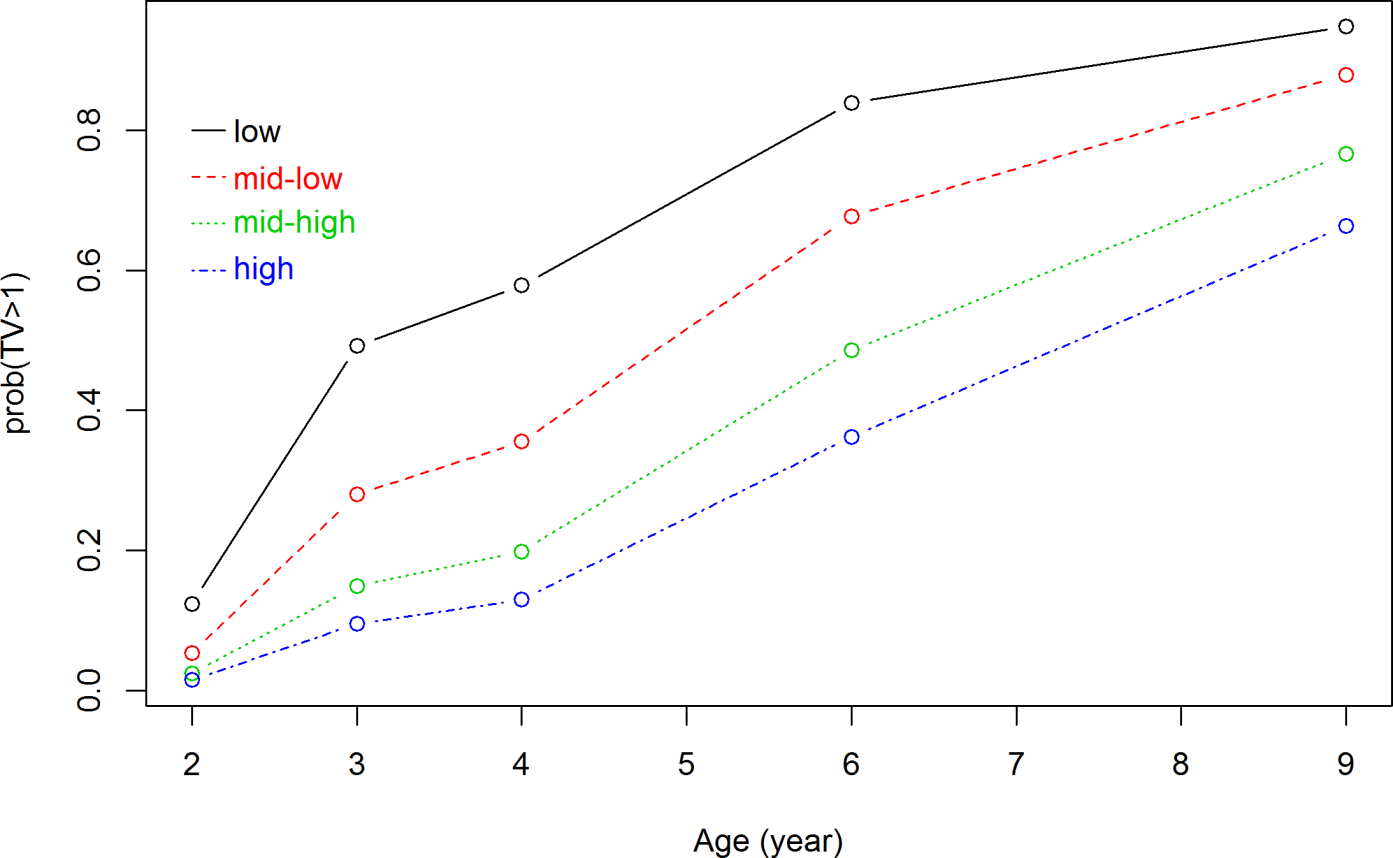
Association between maternal educational level and TV viewing time trajectory. Results are based on generalized logistic mixed model and reflect the probability of watching TV ≥1 hour/day (based on 17805 measurements) in the first 9 years of children of low-, mid-low-, mid-high- and high-educated mother.

**Fig 2:**
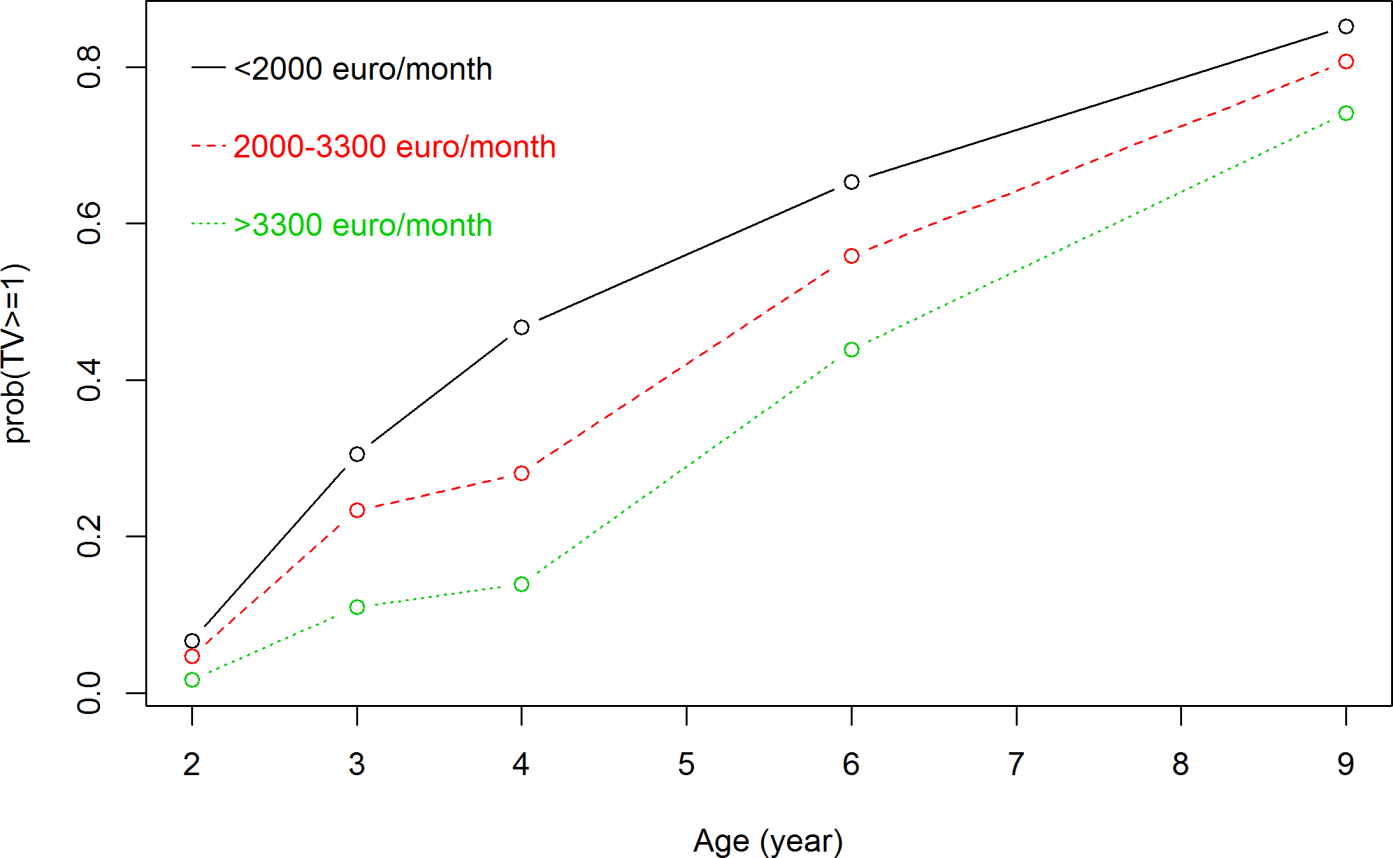
Association between net household income and TV viewing time trajectory. Results are based on generalized logistic mixed model and reflect the probability of watching TV ≥1 hour/day (based on 17805 measurements) in the first 9 years of children from low-, mid- and high-income households.

Because of the missing values of TV viewing time at each measuring time point (12.2% to 28.2%, see Table 1), a total of 17,805 measurements of TV viewing time were available over 5 time points. Results from our GLMM models showed that the probability of TV viewing time ≥1 hour/day increased over time for all socioeconomic subgroups (Figs 1 and 2). Figs 1 and 2 showed all lines increasing from age 2 to age 9. The interaction term between maternal educational level and child age was not significant (p = 0.19), indicating that TV viewing time trajectories were not significantly different for the educational subgroups (Fig 1). The interaction term between net household income and child age was significant (p = 0.01), indicating that children from different income households subgroups showed a different TV viewing time trajectory (Fig 2).

### Associations with TV viewing time ≥2 hours/day

In addition, we evaluated the associations between family SES and child TV viewing time with a secondary outcome variable dichotomized at 2 hours/day. In our study population, there were no children watch TV ≥2 hours/day at age 2 years (Table 1). The results of the cross-sectional analyses were comparable to the previous analyses, although effect estimates (ORs) were larger. Again, children of low educated mothers showed the highest risk watching TV ≥2hours/day (3 years: OR = 8.47, 95% CI: 3.96, 18.10; 4 years: OR = 11.46, 95% CI: 6.42, 20.43; 6 years: OR = 5.36, 95% CI: 3.69, 7.78; 9 years: OR = 5.21, 95%CI: 3.83, 7.09). The OR for TV viewing time ≥2 hours/day for children from low income households was 2.67 (95% CI: 1.45, 4.93) at age 3 years, 3.95 (95% CI: 2.46, 6.33) at age 4 years, 3.18 (95% CI: 2.07, 4.91) at age 6 years and 2.69 (95%CI: 2.03, 3.58) at age 9 years (basic model, S2 Table). Independent associations with child TV viewing time were found for maternal educational level at all ages and for household income except at child age 3 years (full model, S2 Table). In longitudinal analyses, both interaction terms for maternal educational level and net household income (p = 0.41 and p = 0.20, respectively) were not significant (S1 and S2 Figs).

## Discussion

Results from this longitudinal study supported the hypothesis that for children in all the socioeconomic subgroups, TV viewing time increases from age 2 to 9 years. Compared with children from high SES subgroups, children from low SES subgroups were more likely to exceed entertainment-media guidelines (<1 hour/day) at all ages, as expected. The TV viewing trajectories differed significantly between children from high, middle and low income households. However TV viewing trajectories did not differ significantly between children of low-, mid-low-, mid-high- or high-educated mothers, which was not expected.

The finding that number of children engaging in TV viewing time ≥ 1 hour daily increased significantly from age 2 to 9 years confirms previous reports on increases in screen-based entertainment use that occurs during early childhood [18, 27]. An American National Survey reported that the total proportion of young people engaged in TV/video viewing ≥ 2 hours daily was 35.3% for 2–5 years and 49.1% for 6–11 years [27]. In addition, the increase of child TV viewing time we found in all SES groups from age 2 to 9 years is supported by a recent Swedish study, which found that in 7-to-9-year-old school children sedentary behavior increased in both high and low SES groups [19]. Even though children have access to a variety of entertainment media, TV viewing remains the predominant source of children’s screen-based entertainment and sedentary behaviors [28]. Different sedentary behaviors may influence child health differently, however TV viewing is most strongly linked to overweight development. One study reported that TV viewing was associated with overweight; non-school computer usage and reading were not [29]. Another study reported the bi-directional relationship between TV viewing and overweight [30]. Therefore, limiting TV viewing is still a key target for public health intervention in children, especially for preschoolers and school-aged children.

Our finding of associations between family SES and child TV viewing time is in line with studies showing that low SES children more often have a higher TV viewing time than high SES children have [17, 31, 32]. In addition, we found the inverse socioeconomic differences in child TV viewing time at each measuring time points, i.e. age 2, 3, 4, 6 and 9 years. Large socioeconomic differences in TV viewing time occurred as early as age 2 years, and continued: by age 9 years, children of low-educated mothers were four times more likely to be exceeding entertainment-media guidelines (<1 hour/day). These findings differ from results from a study among Greek preschoolers (1–5 years), which found an inverse association between maternal educational level and TV viewing time among children aged 1–2 years but not among children aged 3–5 years [18]. Possible explanations for the discrepancy are that the trajectories of TV viewing time across maternal educational subgroups may vary between countries. Further, TV viewing time was dichotomized at more than or equal to 2 hours per day in the Greek study, which makes the result less prominent for children age under 5 years. Independent associations with child TV viewing time were found in maternal educational level at all ages but not in household income at later ages. One possible explanation for our findings with regard to income, is that children who are in day-care may spend less time watching TV than children who are cared for at home [33]. After the first years (e.g. age 0–4 years in the Netherlands), all children, from both low and high income families, attend primary school. Additional analyses of our data on day-care attendance at the age of 3 years, suggested indeed that children from high income households were more often in day-care ≥ 2 days/week compared to children from low income households. Future studies are recommended to further explore these findings, with regard to the potential explanatory mechanisms. Maternal educational differences in TV viewing remained until age 9 years, which indicates that maternal educational level has an independent role in the socioeconomic differences in child TV viewing behavior.

The present study is a large-scale study assessing socioeconomic inequalities in child TV viewing time trajectory from preschoolers to older school-aged children. Results from our GLMM models showed that the difference in probability of exceeding entertainment-media guidelines (<1 hour/day) between the various educational subgroups remained stable with increasing age, but the differences between the various income subgroups narrowed with increasing age. The socioeconomic differences in TV viewing occurred as early as age 2 years and remained until age 9 years. The association between family SES and child TV viewing time has been found to be mediated by parental attitudes and practices (e.g. availability of media in the bedroom, screen time with parents) [15, 34]. These parenting behaviors offer opportunities for intervention. However more research is needed to investigate the exact impact of these mediating factors in the pathway between SES and child TV viewing. Contrary to the primary outcome of TV≥1 hour/day in the longitudinal associations, the interaction term between child age and net household income was not significant in the sensitivity analyses. In the primary outcome, TV viewing time appeared to differ across socioeconomic subgroups at age 3 and 4 years (Fig 2). During this time period, watching TV no more than 1 hour/day is recommended by all regulations [6, 35, 36]. In our study, the cut-off of TV ≥1 hour/day was more sensitive in capturing the socioeconomic differences in child TV viewing trajectory.

Our results emphasize the need to develop and evaluate interventions for child TV viewing in early childhood. In early childhood socioeconomic differences appeared to be the strongest and interventions most warranted. Early intervention is important to eliminate socioeconomic inequalities in child TV viewing developing. However, not only early childhood but also adolescence may be an important period to intervene. Future studies are recommended to study the development of socioeconomic inequalities in TV viewing time through adolescence, especially taking into consideration the availability of alternative screen-time behaviors. In addition, it is important for policy makers to understand the cumulative effect on children’s health of long-term or short-term exposure to low family SES. However, very few studies have clarified this question and there is a lack of evidence on the influence of children’s TV viewing [37]. Future studies are recommended to study whether periods of low family SES have a greater impact at some life stages than at others. Furthermore, pathways (i.e. parental attitudes and practices, child day-care attendances and availability of alternative screen time sources) underlying the association between family SES and child TV viewing time may be different at different ages, so merit future studies.

### Methodological considerations

Strengths of this study include the large sample of children of different socioeconomic background and the availability of data on repeatedly measured TV viewing time at five time points during childhood. Several limitations of this study should be considered when interpreting the results. First, children with missing data on all five time points of TV viewing time (n = 467) were compared with children that had at least one data point (n = 3965) using Chi-square test for gender, maternal educational level and net household income. Data were more often missing for children with a low maternal educational level (χ^2^ = 376, df = 3, p < 0.001) and children from family with low household income (χ^2^ = 48, df = 2, p < 0.001). This could have led to selection bias, if children with missing data on all five time points watched more TV than the children that we included. Second, potential information bias due to social desirable answering (i.e. the tendency for individuals to overreport desirable behaviors and underreport undesirable behaviors) may have been introduced by the use of parent-reported questionnaires [38]. It is possible that high-educated mothers are more likely to recognize the stigma associated with excessive TV viewing and thus underreport their child’s behavior. Therefore, it is possible that the observed associations underestimated the true associations. Another possible limitation is that information on child television time was derived from 2–4 items in parent-reported questionnaires. Other forms of assessment (e.g. direct observations) are considered to be superior to a few items in a questionnaire [39]. Furthermore, information bias in the outcome variables may have occurred due to the use of different items in questionnaires at each age (see S1 Table). We used the number of days of TV viewing at age 4 years to calculate daily TV viewing time at age 2 and 3 years. This may have introduced information bias. Children at age 2 and 3 may have watched more or less days TV per week than at age 4 years. In this study, maternal educational level and net household income served as indicators of family SES. These variables have been shown to be consistently inversely associated with child TV viewing time [16, 31, 40]. Misclassification of net household income may have occurred since €3300 per month may be a low cut off for high income group, which may lead to an underestimation of income differences in children’s TV viewing time. However, as we have not collected the information on net household income above €3300 per month, this is difficult to ascertain. Furthermore, misclassification of family SES may have occurred after long time follow-up. The indicators of family SES were repeatedly collected at child age 6 years. Compared to the family SES at enrollment, 11% of the mothers had improved their educational level. With regard to net household income, 25.5% of the families changed from lower household income to higher household income and 4.7% family changed from higher household income to lower household income. We repeated the cross-sectional analyses between family SES, measured at child age 6 years, and child TV viewing time at age 6 and at 9 years. The results were comparable to the analyses using family SES at enrollment/child age 2 years, although effect estimates were slightly larger (S3 Table). Our study was conducted in a large sample of Dutch children, therefore future studies are recommended to study the socioeconomic differences in children’s TV viewing trajectory in other large varied population.

## Conclusion

In conclusion, child TV viewing time increases from the preschool period to the school period in all socioeconomic subgroups. During this time, independent inverse effect was found in maternal educational level at all ages but not in net household income. The educational differences between the various educational subgroups remained stable with increasing age, but the differences between household-income groups changed over time. Future studies need to follow-up on the associations between family SES and child TV viewing time when children develop and reach adolescence. Also, underlying pathways associated with family SES and child TV viewing time need to be assessed in, preferably, longitudinal research. Intervention development and healthcare practitioners need to raise awareness among non-highly educated parents about the tracking effect of socioeconomic differences in television viewing time starting from preschool children.

## Supporting information

S1 Table Questionnaire items and calculation of the TV viewing time. (DOCX)

S2 Table Associations of family socioeconomic status with TV viewing (≥2 hours/day) time at each age (n = 3561). (DOCX)

S3 Table Associations of family socioeconomic status (at child age 6 years) with TV viewing time (n = 3561). (DOCX)

S1 Fig Association between maternal educational level and TV viewing time (≥2 hour/day) trajectory. Results are based on generalized logistic mixed model and reflect the probability of watching TV ≥2 hours/day (based on 14244 measurements) from age 3 to 9 years of children of low-, mid-low-, mid-high- and high-educated mother.
(TIFF)

S2 Fig Association between net household income and TV viewing time (≥2 hour/day) trajectory. Results are based on generalized logistic mixed model and reflect the probability of watching TV ≥2 hours/day (based on 14244 measurements) from age 3 to 9 years of children from low-, mid- and high-income households.
(TIFF)
