# Supplementary material for: Socioeconomic differences in children’s television viewing trajectory: A population-based prospective cohort study
Source: PLoS One. 2017 Dec 6;12(12):e0188363. doi: 10.1371/journal.pone.0188363 (PMC5718560; doi:10.1371/journal.pone.0188363)
Supplement: S2 Table — (DOCX) [file pone.0188363.s002.docx]

**Table S2. Associations of family socioeconomic status with TV viewing time (≥2 hours/day) at each age (n=3561)**

|  |  | TV viewing time ≥2 hour/day | | | |
| --- | --- | --- | --- | --- | --- |
|  |  | Age 3 years | Age 4 years | Age 6 years | Age 9 years |
| **Basic model^*^** |  |  |  |  |  |
| Maternal educational level | High | 1 | 1 | 1 | 1 |
|  | Mid-high | **3.30** | **2.03** | **1.67** | **1.37** |
|  |  | **(1.62, 6.71)** | **(1.11, 3.70)** | **(1.17, 2.39)** | **(1.05, 1.77)** |
|  | Mid-low | **6.08** | **4.96** | **2.96** | **2.75** |
|  |  | **(3.07, 12.04)** | **(2.87, 8.56)** | **(2.10, 4.17)** | **(2.13, 3.54)** |
|  | Low | **8.47** | **11.46** | **5.36** | **5.21** |
|  |  | **(3.96, 18.10)** | **(6.42, 20.43)** | **(3.69, 7.78)** | **(3.83, 7.09)** |
|  |  |  |  |  |  |
| Net household income | >€3300/month | 1 | 1 | 1 | 1 |
|  | €2000-3300/month | **2.02** | **2.25** | **1.86** | **1.59** |
|  |  | **(1.23, 3.31)** | **(1.47, 3.46)** | **(1.36, 2.54)** | **(1.25, 2.13)** |
|  | <€2000/month | **2.67** | **3.95** | **3.18** | **2.69** |
|  |  | **(1.45, 4.93)** | **(2.46, 6.33)** | **(2.07, 4.91)** | **(2.03, 3.58)** |
|  |  |  |  |  |  |
| **Full model^**^** |  |  |  |  |  |
| Maternal educational level | High | 1 | 1 | 1 | 1 |
|  | Mid-high | **3.08** | 1.81 | 1.48 | 1.27 |
|  |  | **(1.48, 6.38)** | (0.98, 3.35) | (1.00, 2.19) | (0.97, 1.67) |
|  | Mid-low | **5.51** | **4.16** | **2.45** | **2.46** |
|  |  | **(2.70, 11.25)** | **(2.34, 7.41)** | **(1.62, 3.71)** | **(1.88, 3.22)** |
|  | Low | **7.31** | **8.82** | **4.03** | **4.33** |
|  |  | **(3.20, 16.69)** | **(4.70, 16.58)** | **(2.56, 6.33)** | **(3.10, 6.03)** |
| Net household income | >€3300/month | 1 | 1 | 1 | 1 |
|  | €2000-3300/month | 1.23 | 1.34 | 1.34 | 1.16 |
|  |  | (0.73, 2.08) | (0.85, 2.13) | (0.93, 1.93) | (0.90, 1.49) |
|  | <€2000/month | 1.31 | **1.75** | **1.85** | **1.54** |
|  |  | (0.67, 2.55) | **(1.04, 2.95)** | **(1.11, 3.10)** | **(1.12, 2.10)** |

Table is based on imputed dataset. Bold print indicates statistical significance. Values represent odds ratios and 95% confidence intervals derived from multiple logistic regression analyses.

^*^ Adjusted for confounders (i.e. child's gender and exact age at measurement and maternal age at enrollment).

^**^ Additional adjusted for the other family socioeconomic status indicators.
