# Supplementary material for: Socioeconomic differences in children’s television viewing trajectory: A population-based prospective cohort study
Source: PLoS One. 2017 Dec 6;12(12):e0188363. doi: 10.1371/journal.pone.0188363 (PMC5718560; doi:10.1371/journal.pone.0188363)
Supplement: S3 Table — (DOCX) [file pone.0188363.s003.docx]

**Table S3. Associations of family socioeconomic status (at child age 6 years) with TV viewing time (n=3561)**

|  |  | TV viewing time ≥1 hour/day | |
| --- | --- | --- | --- |
|  |  | Age 6 years | Age 9 years |
| **Basic model^*^** |  |  |  |
| Maternal educational level^***^ | High | 1 | 1 |
|  | Mid-high | **1.30** | **1.41** |
|  |  | **(1.09, 1.55)** | **(1.14, 1.75)** |
|  | Mid-low | **2.44** | **1.64** |
|  |  | **(2.01, 2.97)** | **(1.30, 2.06)** |
|  | Low | **3.82** | **3.69** |
|  |  | **(2.76, 5.28)** | **(2.47, 5.53)** |
| Net household income^***^ | >€3300/month | 1 | 1 |
|  | €2000-3300/month | **1.35** | **1.31** |
|  |  | **(1.13, 1.61)** | **(1.05, 1.62)** |
|  | <€2000/month | **1.77** | **1.68** |
|  |  | **(1.37, 2.27)** | **(1.22, 2.31)** |
| **Full model^**^** |  |  |  |
| Maternal educational level | High | **1** | **1** |
|  | Mid-high | **1.27** | **1.30** |
|  |  | **(1.06, 1.53)** | **(1.06, 1.61)** |
|  | Mid-low | **2.35** | **2.51** |
|  |  | **(1.90, 2.92)** | **(1.93, 3.26)** |
|  | Low | **3.64** | **3.88** |
|  |  | **(2.54, 5.23)** | **(2.29, 6.59)** |
| Net household income | >€3300/month | **1** | **1** |
|  | €2000-3300/month | 0.99 | 0.97 |
|  |  | (0.82, 1.20) | (0.77, 1.23) |
|  | <€2000/month | 1.21 | 1.11 |
|  |  | (0.92, 1.58) | (0.79, 1.56) |

Table is based on imputed dataset. Bold print indicates statistical significance. Values represent odds ratios and 95% confidence intervals derived from multiple logistic regression analyses.

^*^ Adjusted for confounders (i.e. child's gender and exact age at measurement and maternal age at enrollment).

^**^ Additional adjusted for the other family socioeconomic status indicators.

^***^ Maternal educational level and net household income were collected at child age 6 years.
