# Supplementary figures and images for: Socioeconomic differences in children’s television viewing trajectory: A population-based prospective cohort study
Source: PLoS One. 2017 Dec 6;12(12):e0188363. doi: 10.1371/journal.pone.0188363 (PMC5718560; doi:10.1371/journal.pone.0188363)

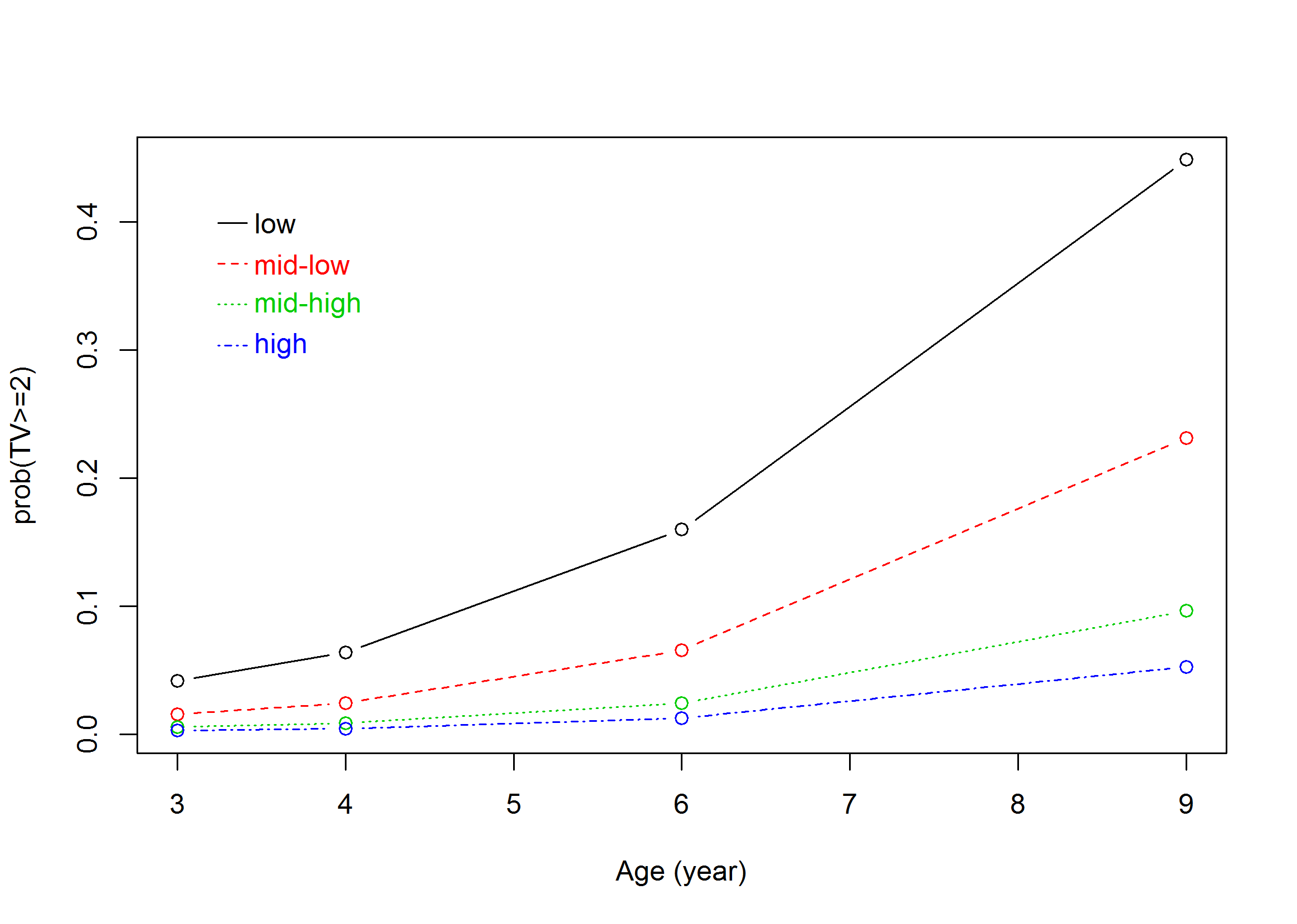

Supplement: S1 Fig — Results are based on generalized logistic mixed model and reflect the probability of watching TV ≥2 hours/day (based on 14244 measurements) from age 3 to 9 years of children of low-, mid-low-, mid-high- and high-educated mother. (TIFF) [file pone.0188363.s004.tiff]

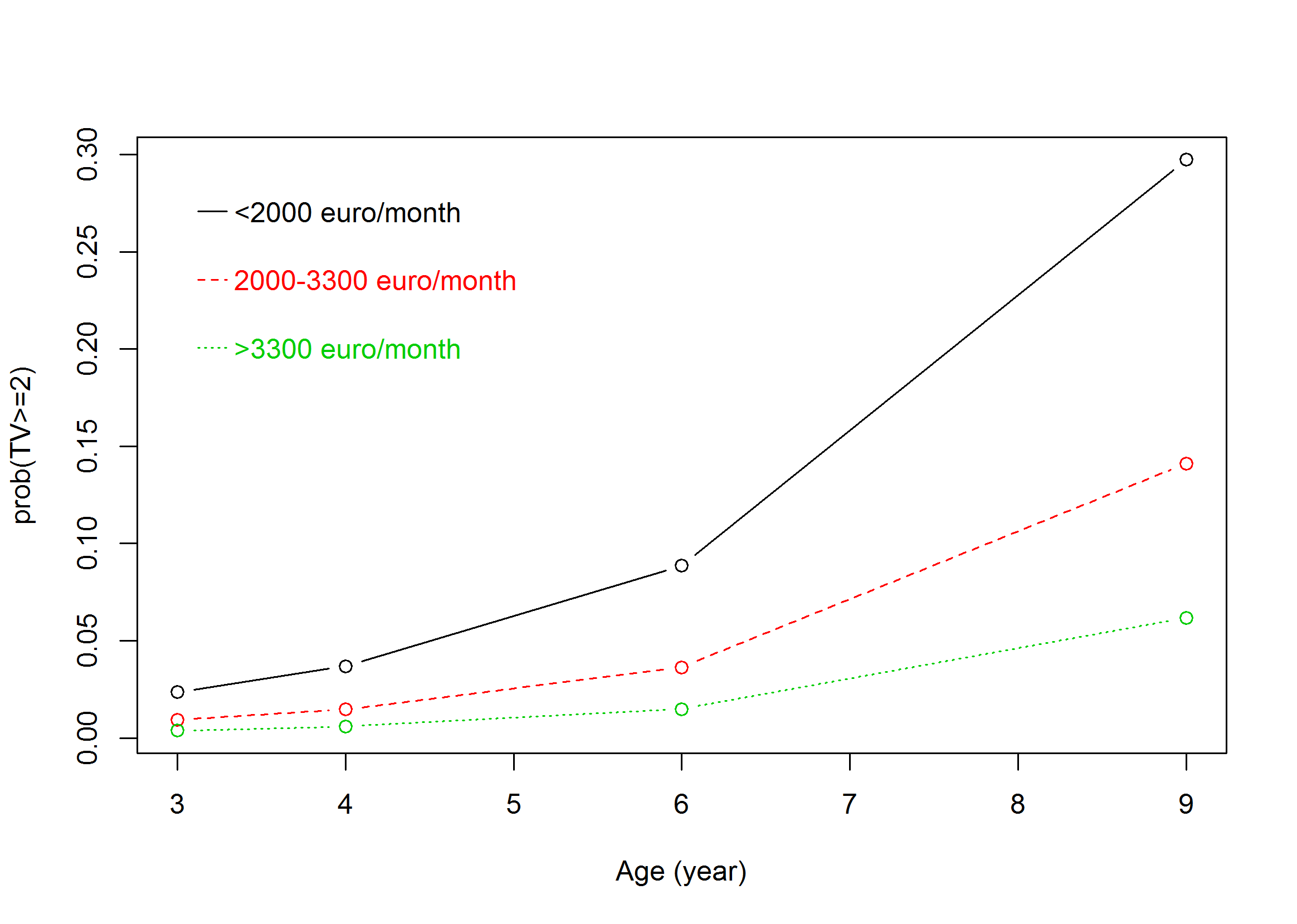

Supplement: S2 Fig — Results are based on generalized logistic mixed model and reflect the probability of watching TV ≥2 hours/day (based on 14244 measurements) from age 3 to 9 years of children from low-, mid- and high-income households. (TIFF) [file pone.0188363.s005.tiff]
